# Optimized Scheduling Technique of Null Subcarriers for Peak Power Control in 3GPP LTE Downlink

**DOI:** 10.1155/2014/279217

**Published:** 2014-04-17

**Authors:** Soobum Cho, Sang Kyu Park

**Affiliations:** ^1^Department of Electrical Engineering, Stanford University, Stanford, CA 94305, USA; ^2^Department of Electronics and Computer Engineering, Hanyang University, Seoul 133-791, Republic of Korea

## Abstract

Orthogonal frequency division multiple access (OFDMA) is a key multiple access technique for the long term evolution (LTE) downlink. However, high peak-to-average power ratio (PAPR) can cause the degradation of power efficiency. The well-known PAPR reduction technique, dummy sequence insertion (DSI), can be a realistic solution because of its structural simplicity. However, the large usage of subcarriers for the dummy sequences may decrease the transmitted data rate in the DSI scheme. In this paper, a novel DSI scheme is applied to the LTE system. Firstly, we obtain the null subcarriers in single-input single-output (SISO) and multiple-input multiple-output (MIMO) systems, respectively; then, optimized dummy sequences are inserted into the obtained null subcarrier. Simulation results show that Walsh-Hadamard transform (WHT) sequence is the best for the dummy sequence and the ratio of 16 to 20 for the WHT and randomly generated sequences has the maximum PAPR reduction performance. The number of near optimal iteration is derived to prevent exhausted iterations. It is also shown that there is no bit error rate (BER) degradation with the proposed technique in LTE downlink system.

## 1. Introduction

The fields of mobile communication techniques have been rapidly developed in recent decades. One of the development results is the 3rd generation partnership project (3GPP) long term evolution (LTE), which has been deployed all over the world. Downlink transmission of the LTE is based on the use of multiple access technology: orthogonal frequency division multiple access (OFDMA), which is a modification of orthogonal frequency division multiplexing (OFDM) for the multiple access [1, 2]. Recent advances of digital signal processing (DSP) technique have accelerated the popularity of the OFDM. The technique has a lot of tolerances to frequency selective fading and multipath interference; therefore it has been adapted to numerous international standards for wired and wireless communication systems such as very-high-bit-rate digital subscriber line (VDSL) [3], power line communication (PLC) [4], wireless local area network (WLAN) [5], and ultrawideband (UWB) [6]. It is also attracting a lot of interest in visible light communication (VLC) and optical wireless communications [7].

However, together with its advantages, still some challenging issues remain for the OFDM access technology design. One of the major drawbacks is high peak-to-average power ratio (PAPR) of transmitted signals. Therefore, the detection efficiency of the OFDM receiver is very sensitive to the nonlinear devices such as digital-to-analog converter (DAC) and high power amplifier (HPA). That may severely diminish the system performance because of the detection efficiency degradation and induced spectral regrowth. Most of the transmitters of wireless communication systems employ the HPA to obtain sufficient transmit power. The HPA usually operates near the saturation region to achieve the maximum output power efficiency; thus the memoryless nonlinear distortions occur in the communication channels due to the high PAPR of the input signals. If the HPA does not operate within linear region with power back-off (PBO), it is difficult to keep the out-of-band power below the specified limits. This situation leads to very inefficient amplification and expensive transmitters [8]. Therefore, it is important to do research on the characteristics of the PAPR including its reduction in order to use the features of the OFDM.

To deal with the PAPR problem, various approaches have been proposed such as deliberate clipping [9], partial transmit sequence (PTS) [10], selected mapping (SLM) [11], interleaving [12], coding [13], tone reservation (TR) [14], active constellation extension (ACE) [15], and dummy sequence insertion (DSI) [16]. These techniques reduce the PAPR by the trade-off among signal power, data rate, system complexity, and bit error rate (BER) performance. The DSI scheme can be simply implemented by scheduling some dummy sequences which are used only for PAPR reduction. However, the large usage of additional subcarriers for the dummy sequences can directly cause the reduction of transmission efficiency. The number of dummy sequences needed for the desired PAPR reduction level depends on the feature of the communication systems. In [17], the number of unused subcarriers was calculated in the LTE single-input single-output (SISO) system and it was used for the dummy sequences with cyclic shifted sequences scheme. However, the PAPR reduction performance was still high, since dummy sequences were not optimally scheduled. Furthermore, BER performances were not compared with the conventional LTE system even though the BER is a very important aspect.

In this paper, the null subcarriers for the dummy sequences are derived in LTE SISO, 2 × 2, and 4 × 4 multiple-input multiple-output (MIMO) systems, respectively, and transmission efficiencies are calculated. The optimal design of the dummy sequences is derived and it is scheduled to control the peak power. Finally, the simulation result shows the comparison of the BER performances which demonstrates that the proposed method can reduce the PAPR considerably without BER performance degradation.

This paper is organized as follows.  Section 2 describes the PAPR and complementary cumulative distribution function (CCDF) definitions. In Section 3, the null subcarriers of the LTE SISO, 2 × 2, and 4 × 4 MIMO systems are derived.  Section 4 shows the simulation results and analyzes the performances. Finally, Section 5 offers our conclusions and future works.

## 2. PAPR and CCDF Definitions

Several drawbacks arise in OFDM, the most severe of which is the highly nonconstant envelope of the transmitted signals, that is, the PAPR, making the OFDM very sensitive to nonlinear components in the transmission path. The use of HPA can be one of the solutions. However, owing to cost, design, and, most importantly, power efficiency considerations, the HPA cannot resolve the dynamics of the transmitted signal. A clipping method inevitably cuts off the signal at some point, which causes additional in-band distortion and adjacent channel interference. The power efficiency penalty is certainly the major obstacle in implementing OFDM systems in low-cost applications. Moreover, in power limited regimes determined by regulatory bodies, the average power is reduced in comparison to single-carrier systems. The main goal of peak power control is to diminish the influence of high peaks in transmit signals on the performance of the transmission system. The PAPR of the transmit signal can be defined as

(1) PAPR=max⁡0≤k≤NJ−1⁡|xk|2E[|xk|2],

where *E*[·] denotes mathematical expectation.

The CCDF which denotes the probability that the PAPR of a data block exceeds a given threshold is one of the most frequently used performance measures for PAPR reduction techniques. If the number of subcarriers is large enough, magnitudes of real and imaginary parts of output signal have Gaussian distribution with mean of zero and variance of 1/2 by central limit theorem. Thus, the amplitude of the OFDM signal follows Rayleigh distribution while the power distribution of OFDM signal is central chi-square distribution with two degrees of freedom and a mean of zero. The CCDF of the PAPR of a data block with Nyquist rate sampling is derived as

(2) Pr(PAPR>PAPR0)=1−(1−exp⁡⁡(−PAPR0))N,

where PAPR_0_ is the threshold PAPR. This expression assumes that the *N* time domain signal samples are mutually independent and uncorrelated. However, when oversampling is applied, the assumption is no longer valid. The CCDF of the PAPR for *N* subcarriers with oversampling is given by

(3) Pr(PAPR>PAPR0)=1−(1−exp⁡⁡(−PAPR0))αN,

where *α* is a certain number expressing the effect of oversampling.

## 3. Calculation of the Null Subcarriers of the LTE Downlink System

### 3.1. Frame Structure

**Figure 1:**
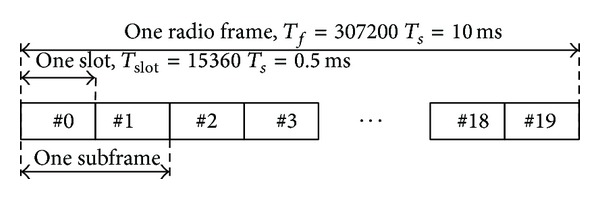
Frame structure type 1 [18].

There are two radio frame structures for LTE, that is, frame structure type 1 (FS1) for full and half duplex frequency division duplex (FDD) and frame structure type 2 (FS2) for time division duplex (TDD). This paper focuses on FDD. In FDD, because uplink and downlink transmissions are separated in the frequency domain, the frame structure is the same in the uplink and downlink in terms of frame, subframe, and slot duration. FS1 is shown in Figure 1.

The size of various fields in the time domain is expressed as a number of time units, *T*_*s*_. This structure consists of ten 1 ms subframes, each composed of two 0.5 ms slots (*T*_slot_ = 15360*T*_*s*_ = 0.5 ms) for a total duration of 10 ms (*T*_*f*_ = 307200*T*_*s*_ = 10 ms).

### 3.2. Downlink Physical Resource Elements

**Figure 2:**
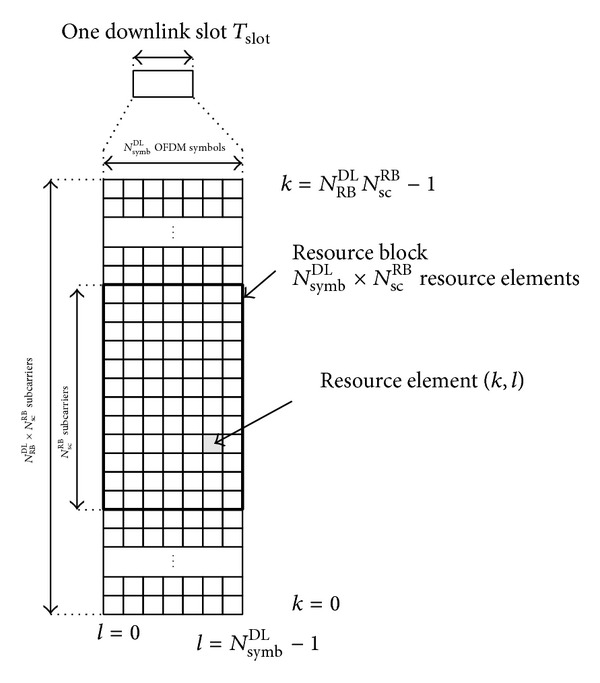
Downlink resource grid [18].

One symbol on one subcarrier is defined as the resource element, which is the smallest time-frequency unit used for downlink transmission. A group of twelve contiguous subcarriers in frequency and one slot in time is called a resource block (RB) [19], which is shown in Figure 2.

A physical RB consists of *N*_symb_^DL^ × *N*_sc_^RB^ resource elements, where *N*_symb_^DL^ is the number of symbols per slot and *N*_sc_^RB^ is the number of subcarriers per RB.

One downlink slot using the normal cyclic prefix (CP) length contains seven symbols. Variations on this configuration for FS1 are summarized in Table 1. The CP is chosen to be slightly longer than the longest expected delay spread in the radio channel.

### 3.3. Null Subcarriers in PDSCH of SISO, 2 × 2, and 4 × 4 MIMO Systems

Firstly, the number of null subcarriers per frame, *N*_spaces,1×1_^FRM^, is calculated in the physical downlink shared channel (PDSCH) of the LTE SISO system [17]. The number of data per RB, *N*_data,1×1_^RB^, can be obtained as

(4) Ndata,1×1RB=NsymbDL×NscRB−NpilotRB−Nu-pilot,1×1RB=7×12−4−0=80,

where *N*_symb_^DL^ is the number of symbols per slot, which is 7 in normal CP, *N*_sc_^RB^ is the number of subcarriers per RB, which is 12, *N*_pilot_^RB^ is the number of reference signals (RSs) per RB, which is 4, and *N*_u-pilot,1×1_^RB^ is the number of RSs used in other antenna ports per RB, which is 0 in SISO system. The number of data per subframe, *N*_data,1×1_^S-FRM^, can be calculated as

(5) Ndata,1×1S-FRM=Ndata,1×1RB×2−NPDCCH,1×1RB=80×2−34=126,

where *N*_PDCCH,1×1_^RB^ is the number of symbols for the physical downlink control channel (PDCCH) per RB, which is 34 in SISO system, and two means there are two slots in one subframe. The number of data per frame, *N*_data,1×1_^FRM^, can be calculated as

(6) Ndata,1×1FRM=Ndata,1×1S-FRM×NRBLEN×Nmod⁡ODR×TBCR=126×5×2×13=420,

where *N*_RB_^LEN^ (≤*N*_RB_^DL^) is the number of RBs, assumed to be five in this paper. *N*_RB_^DL^ is the maximum number of RBs for fixed transmission bandwidth, which is 25 at the 5 MHz bandwidth. *N*_mod⁡_^ODR^ is the modulation order, which is 2, because this paper assumes quadrature phase shift keying (QPSK) modulation. TB_CR_ is the turbo coding rate, which is defined as 1/3 in PDSCH. Therefore, the number of data created in the data source step, *N*_data,1×1_^DS^, can be calculated as

(7) Ndata,1×1DS=(Ndata,1×1FRM−NCW,1×1×NchksumCRC−NPBCH,1×1FRM− NS-SSFRM−NP-SSFRM)×(NCW,1×1)−1=420−1×24−23−6−61=361,

where *N*_CW,1×1_ is the number of code words in SISO system, which is 1, *N*_chksum_^CRC^ is the number of cyclic redundancy check (CRC) checksums, which is 24 bits, *N*_PBCH,1×1_^FRM^ is the number of symbols for the physical broadcast channel (PBCH) per frame in SISO, *N*_S-SS_^FRM^ is the number of secondary synchronization signals per frame, and *N*_P-SS_^FRM^ is the number of primary synchronization signals per frame. The number of data after the CRC encoding, *N*_data,1×1_^CRC^, can be calculated as

(8) Ndata,1×1CRC=Ndata,1×1DS+NchksumCRC=361+24=385,

where *N*_chksum_^CRC^ is the number of CRC checksums. Then, the number of data after turbo encoding, *N*_data,1×1_^TURBO^, can be calculated as

(9) Ndata,1×1TURBO=TableSearch(Ndata,1×1CRC)×3+12=492×3+12=1188,

where TableSearch(·) is the turbo encoding table. The number of data after QPSK modulation and layer mapping, *N*_data,1×1_^MOD^, can be calculated as

(10) Ndata,1×1MOD=Ndata,1×1TURBONmod⁡ODR=11882=594,

where *N*_mod⁡_^ODR^ is the modulation order. Finally, the number of null subcarriers after the resource element mapping step, *N*_spaces,1×1_^FRM^, can be calculated as

(11) Nspaces,1×1FRM=NdataN-FRM−Ndata,1×1MOD=630−594=36,

where *N*_data_^N-FRM^ is the number of data in the normal frame of SISO system. Therefore, there are 36 null subcarriers following the resource element mapping step in PDSCH of the LTE SISO system and it can be used for inserting dummy sequences.

Secondly, the number of null subcarriers per frame is obtained in the PDSCH of the LTE 2 × 2 MIMO system. The number of data per RB in 2 × 2 MIMO, *N*_data,2×2_^RB^, can be calculated as

(12) Ndata,2×2RB=NsymbDL×NscRB−NpilotRB−Nu-pilot,2×2RB=7×12−4−4=76,

where *N*_u-pilot,2×2_^RB^ is the number of RSs used in other antenna ports per RB, which is 4 in 2 × 2 MIMO system. The number of data per subframe, *N*_data,2×2_^S-FRM^, is obtained as

(13) Ndata,2×2S-FRM=Ndata,2×2RB×2−NPDCCH,2×2RB=76×2−32=120,

where *N*_PDCCH,2×2_^RB^ is the number of symbols for the PDCCH per RB in 2 × 2 MIMO system. The number of data per frame in 2 × 2 MIMO, *N*_data,2×2_^FRM^, is derived as

(14) Ndata,2×2FRM=NTX×Ndata,2×2S-FRM×NRBLEN×Nmod⁡ODR×TBCR=2×120×5×2×13=800,

where *N*_TX_ is the number of antenna which is 2 in 2 × 2 MIMO system. Therefore, the number of data created in the data source step, *N*_data,2×2_^DS^, can be obtained as

(15) Ndata,2×2DS=(Ndata,2×2FRM−NCW,2×2×NchksumCRC− NPBCH,2×2FRM−NS-SSFRM−NP-SSFRM)×(NCW,2×2)−1=800−1×24−22−6−61=742,

where *N*_CW,2×2_ is the number of code words in 2 × 2 MIMO system, which is 1, and *N*_PBCH,2×2_^FRM^ is the number of symbols for PBCH per frame in 2 × 2 MIMO, which is 22. The number of data after the CRC encoding in 2 × 2 MIMO, *N*_data,2×2_^CRC^, can be calculated as

(16) Ndata,2×2CRC=Ndata,2×2DS+NchksumCRC=742+24=766.

Then, the number of data after turbo encoding, *N*_data,2×2_^TURBO^, can be derived as

(17) Ndata,2×2TURBO=TableSearch(Ndata,2×2CRC)×3+12=768×3+12=2316.

The number of data after modulation and layer mapping, *N*_data,2×2_^MOD^, is calculated as

(18) Ndata,2×2MOD=Ndata,2×2TURBO/Nmod⁡ODRNTX=2316/22=579.

Finally, the number of null subcarriers after the resource element mapping step, *N*_spaces,2×2_^FRM^, can be obtained as

(19) Nspaces,2×2FRM=Ndata,2×2N-FRM−Ndata,2×2MOD=600−579=21,

where *N*_data,2×2_^N-FRM^ is the number of data in the normal frame of 2 × 2 MIMO system. Therefore, there are 21 null subcarriers.

Thirdly, *N*_spaces,4×4_^FRM^, is derived in the PDSCH of the LTE 4 × 4 MIMO system. The number of data per RB is derived as

(20) Ndata,4×4RB=NsymbDL×NscRB−NpilotRB−Nu-pilot,4×4RB=7×12−4−8=72,

where *N*_u-pilot,4×4_^RB^ is the number of RSs used in other antenna ports per RB in 4 × 4 MIMO system. The number of data per subframe, *N*_data,4×4_^S-FRM^, can be obtained as

(21) Ndata,4×4S-FRM=Ndata,4×4RB×2−NPDCCH,4×4RB=72×2−28=116,

where *N*_PDCCH,4×4_^RB^ is 28 in 4 × 4 MIMO system. The number of data per frame, *N*_data,4×4_^FRM^, in 4 × 4 MIMO can be obtained as

(22) Ndata,4×4FRM=NTX×Ndata,4×4S-FRM×NRBLEN×Nmod⁡ODR×TBCR=4×116×5×2×13=1546,

where *N*_TX_ is 4. Therefore, the number of data created in the data source step, *N*_data,4×4_^DS^ in 4 × 4 MIMO, is derived as

(23) Ndata,4×4DS=(Ndata,4×4FRM−NCW,4×4×NchksumCRC− NPBCH,4×4FRM−NS-SSFRM−NP-SSFRM)×(NCW,4×4)−1=1546−2×24−20−6−61=733,

where *N*_CW,4×4_ is 2 and *N*_PBCH,4×4_^FRM^ is 20 in 4 × 4 MIMO system. The number of data after the CRC encoding, *N*_data,4×4_^CRC^, is obtained as

(24) Ndata,4×4CRC=Ndata,4×4DS+NchksumCRC=733+24=757.

Then, the number of data after turbo encoding, *N*_data,4×4_^TURBO^, in 4 × 4 MIMO system can be obtained as

(25) Ndata,4×4TURBO=TableSearch(Ndata,4×4CRC)×3+12=768×3+12=2316.

The number of data after QPSK modulation and layer mapping, *N*_data,2×2_^MOD^, can be derived as

(26) Ndata,4×4MOD=Ndata,4×4TURBO/Nmod⁡ODRNTX=2316/24=297.

Therefore, *N*_spaces,4×4_^FRM^ in 4 × 4 MIMO can be derived as

(27) Nspaces,4×4FRM=Ndata,4×4N-FRM−Ndata,4×4MOD=300−297=3,

where *N*_data,4×4_^N-FRM^ is the number of data in the normal frame of 4 × 4 MIMO system. Finally, there are 3 null subcarriers following the resource element mapping step in PDSCH of the LTE 4 × 4 MIMO system.

We have derived the null subcarriers for SISO, 2 × 2, and 4 × 4 MIMO systems, respectively. Since this paper focuses on SISO LTE system, we assume that the maximum dummy subcarrier is 36, which does not decrease the transmission efficiency. When MIMO LTE system is applied, we may sacrifice some decrease in transmission efficiency, which can be defined as

(28) transmission efficiency=N−(36−NspacesFRM)N×100[%],

where *N* is the number of subcarriers and *N*_spaces_^FRM^ is the number of null subcarriers. Therefore, the transmission efficiencies of the 2 × 2 and 4 × 4 MIMO LTE systems are 97% and 94%, respectively, when *N* is 512.

## 4. Simulation Results and Discussion

In the proposed scheme, there is a trade-off between the type and pattern of dummy sequence and the iteration time for the cyclic shift. Therefore, consideration of these elements is an important aspect of PAPR reduction performance and suitable system complexity. In this section, we find the near optimum values for the DSI method, the ratio of dummy sequence for the null subcarriers, and the number of iterations by various simulation results. The simulations are performed under the 3GPP LTE physical layer standard [18, 19].  Table 2 lists the parameters of our simulations.

For the suitable DSI, we compare the PAPR reduction performances of the well-known DSI methods. The DSI methods are briefly introduced as follows.

- Method 1: complementary sequences and correlation sequences corresponding to the first bits of each partitioned subblock are inserted as dummy sequences before the inverse fast Fourier transform (IFFT) stage [16].
- Method 2: WHT is inserted as a dummy sequence before the IFFT stage [20].
- Method 3: time-frequency domain swapping algorithm and flipping technique are used to optimize the phase of dummy sequences [21].
- Method 4: every initial dummy sequence is “0” and employs bit flipping method to generate dummy sequences for next branch [22].
- Method 5: the total sequences consist of *L* length data sequences and *M* length dummy sequences. A dummy bit is inserted at the end of the sequences for binary addition between adjacent bits (*L* + *M*)/2 + 1 and *L* + *M*. At the same time, the *N*-point IFFT block is divided into two sub-IFFT blocks to reduce the IFFT complexity [23].
- Method 6: a partial DSI method is a combination of the DSI and the PTS. The original data sequences are partitioned and zero padded, and a “0” or “1” dummy sequence is inserted into each subblock. The time domain waveforms are summed after IFFT, and the sequence with the lowest PAPR is selected and transmitted [24].

**Figure 3:**
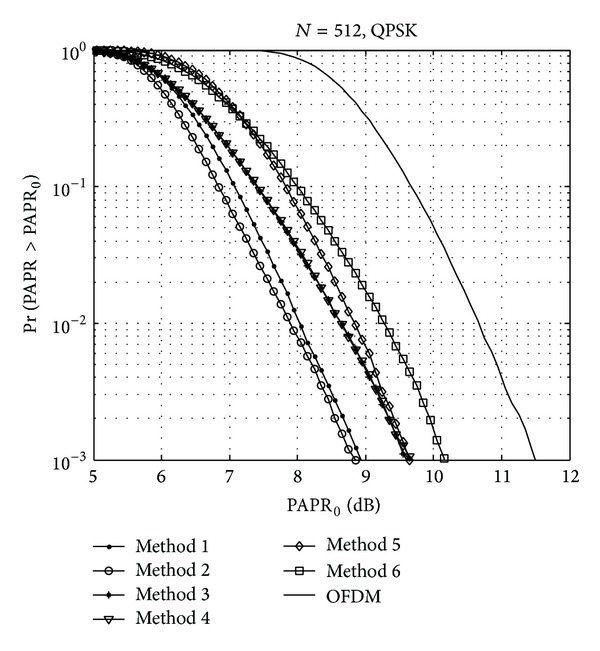
CCDF comparison of DSI methods.

Figure 3 shows the CCDF comparison among the six kinds of the DSI methods. Considering PAPR reduction performance, we can conclude that Method 2, that is, WHT sequences, is the best choice for the dummy sequence.

**Figure 4:**
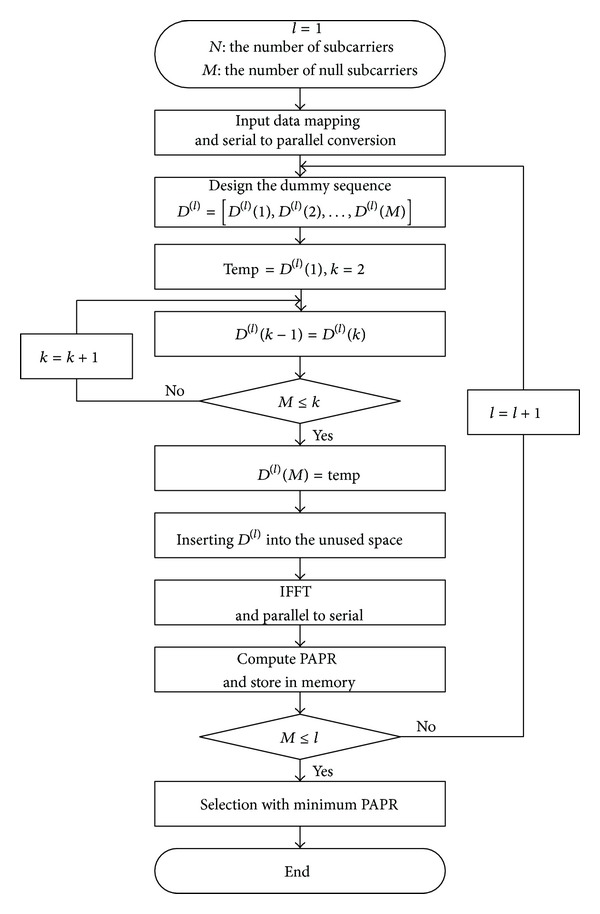
Flow chart of scheduling the null subcarriers for peak power reduction.

As we analyzed in Section 3, there are 36 null subcarriers following the resource element mapping step in the PDSCH of the LTE SISO system. The essence of the proposed scheme is making full use of the null subcarriers, which have to be designed for optimal PAPR reduction performance. It is derived that WHT is the most suitable for the dummy sequences. Since the length of WHT is 2^*n*^ = 2,4, 8,16,32 ≤ 36, (*n* = 1,2, 3,4, 5), the 36 null subcarriers are partitioned into two parts. The first part is for inserting WHT and the second part is for inserting randomly generated sequences with {−1, 1} elements as a dummy sequence. Then, 36 null subcarriers are cyclic shifted with *l* time iterations to find the minimum PAPR.  Figure 4 shows the flow chart of the proposed method.

Since PAPR performance is affected by the patterns of the WHT, the PAPR performances of the proposed method are compared by the ratio of the WHT and randomly generated sequence. The five patterns of the WHT and randomly generated sequence can be in the ratio of 32/4, 16/20, 8/28, 4/32, and 2/34.

**Figure 5:**
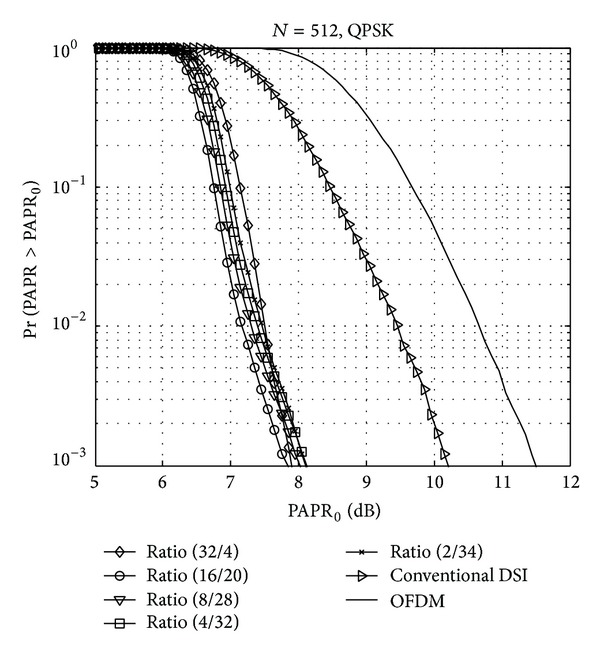
CCDF comparison as a function of WHT and randomly generated sequence ratio.

Figure 5 shows the CCDF of the PAPR of the five kinds of null subcarrier design with full iteration (*l* = *M*). Since the ratio of 16/20 has the best PAPR reduction performance, we can conclude that the ratio of 16/20 is the optimal choice for the proposed method.

**Figure 6:**
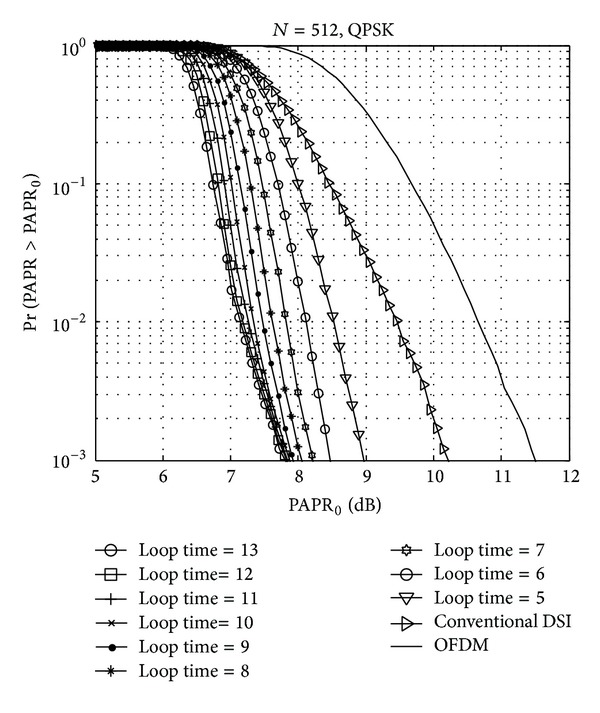
CCDF comparison over the number of iterations.

In order to approach a more efficient PAPR reduction within the limited null subcarriers, cyclic shifting is used with WHT sequences. Multiple iteration operations for the cyclic shifting, however, cause the high computational complexity of the LTE system. Therefore, we need to consider the cyclic shift loop times to approach the minimum computational complexity. The PAPR performances of various cyclic shift loop times are compared in Figure 6. Obviously, the bigger cyclic shift loop time has more PAPR reduction performance. Nevertheless, PAPR performances are saturated to about 7.9 dB. Therefore, we can conclude that 9 may be the nearly optimal number of iterations.

**Figure 7:**
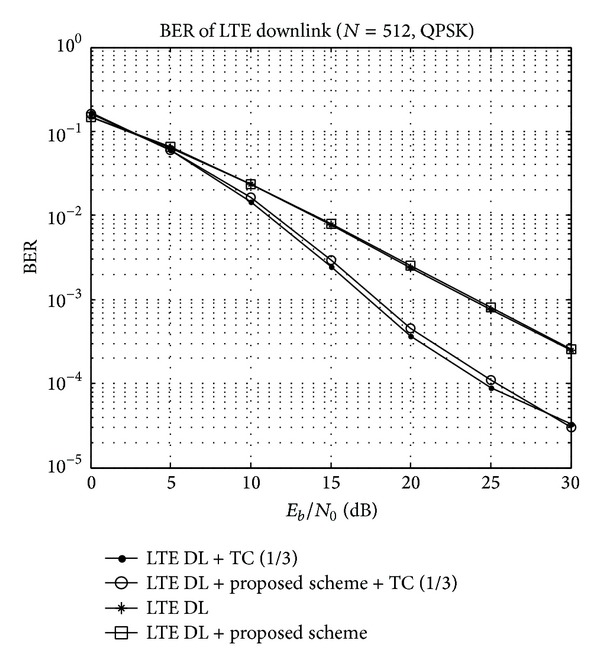
BER performance of the proposed scheme and LTE system.

In addition to the PAPR comparison, we examine the BER performance of the proposed method in the LTE downlink system. Simulation is performed under Rayleigh fading channel, and the turbo coding is used with a coding rate of *R* = 1/3. As shown in Figure 7, the conventional LTE downlink system and the proposed method have nearly the same BER performance. Therefore, the proposed scheme can reduce the PAPR for the LTE downlink system considerably without the degradation of the BER performance.

## 5. Conclusions

This paper proposed a novel DSI scheme for the LTE downlink system. For the application of the DSI to LTE system, the null subcarriers were obtained in LTE SISO, 2 × 2, and 4 × 4 MIMO systems, respectively, and each transmission efficiency was calculated. The dummy sequence was designed by scheduling the ratio between WHT and random sequences. The number of near optimal iteration and BER performances were derived, which showed that exhausted iterations could be prevented and proposed DSI can reduce PAPR without BER degradation.

The future works will derive the number of subcarriers in LTE-Advanced and 8 × 8 MIMO systems. To overcome the PAPR reduction performance with the limited null subcarrier, the other dummy sequences will be applied. In addition, new algorithm will be researched to reduce the iteration time or eliminate it completely for the more realistic system.

## Figures and Tables

**Table 1:** Physical resource block parameters [18].

Configuration		*N*_sc_^RB^	*N*_symb_^DL^
Normal cyclic prefix	Δ*f* = 15 kHz	12	7
Extended cyclic prefix	Δ*f* = 15 kHz	12	6
Extended cyclic prefix	Δ*f* = 7.5 kHz	24	3

**Table 2:** Parameters of the computer simulations.

Parameter	Value
Carrier frequency *f*_0_	2140 MHz
Channel bandwidth	2.5 MHz
FFT size	512
Duplex mode	FDD
Cyclic shift	Normal
Modulation type	QPSK
Doppler frequency	119 MHz (velocity = 60 Km/h)
CRC	24 bit
Forward error correction (FEC)	1/3 turbo coding
Number of dummy bit	36
